# Relationship of Non-Invasive Arterial Stiffness Parameters with 10-Year Atherosclerotic Cardiovascular Disease Risk Score in Post-COVID-19 Patients—The Results of a Cross-Sectional Study

**DOI:** 10.3390/life14091105

**Published:** 2024-09-02

**Authors:** Danuta Loboda, Beata Sarecka-Hujar, Marta Nowacka-Chmielewska, Izabela Szoltysek-Boldys, Wioleta Zielinska-Danch, Michal Gibinski, Jacek Wilczek, Rafal Gardas, Mateusz Grabowski, Mateusz Lejawa, Andrzej Malecki, Krzysztof S. Golba

**Affiliations:** 1 Department of Electrocardiology and Heart Failure, Medical University of Silesia in Katowice, 40-635 Katowice, Poland; mgibinski@sum.edu.pl (M.G.); jwilczek@sum.edu.pl (J.W.); rafal.gardas@sum.edu.pl (R.G.); kgolba@sum.edu.pl (K.S.G.); 2 Department of Basic Biomedical Science, Faculty of Pharmaceutical Sciences in Sosnowiec, Medical University of Silesia in Katowice, 41-200 Sosnowiec, Poland; bsarecka-hujar@sum.edu.pl; 3 Laboratory of Molecular Biology, Institute of Physiotherapy and Health Sciences, Academy of Physical Education in Katowice, 40-065 Katowice, Poland; m.nowacka@awf.katowice.pl (M.N.-C.); m.grabowski@awf.katowice.pl (M.G.); mateusz.lejawa@sum.edu.pl (M.L.); a.malecki@awf.katowice.pl (A.M.); 4 Department of General and Inorganic Chemistry, Faculty of Pharmaceutical Sciences in Sosnowiec, Medical University of Silesia in Katowice, 41-200 Sosnowiec, Poland; iboldys@sum.edu.pl (I.S.-B.); wzdanch@sum.edu.pl (W.Z.-D.); 5 Department of Pharmacology, Faculty of Medical Sciences in Zabrze, Medical University of Silesia in Katowice, 41-808 Zabrze, Poland

**Keywords:** arterial stiffness, atherosclerotic cardiovascular diseases, COVID-19 disease, risk prediction, Systematic Coronary Risk Evaluation 2 algorithm

## Abstract

This study evaluated the relationship of non-invasive arterial stiffness parameters with an individual 10-year risk of fatal and non-fatal atherosclerotic cardiovascular disease (ASCVD) events in the cohort post-coronavirus disease 2019 (COVID-19). The study group included 203 convalescents aged 60.0 (55.0–63.0) and 115 (56.7%) women. The ASCVD risk was assessed as low to moderate to very high based on medical history (for 62 participants with pre-existing ASCVD/diabetes/chronic kidney disease in the entire cohort) or calculated in percentages using the Systemic Coronary Risk Evaluation 2 (SCORE2) algorithm based on age, sex, smoking status, systolic blood pressure (BP), and non-high-density lipoprotein cholesterol (for 141 healthy participants). The stiffness index (SI) and reflection index (RI) measured by photoplethysmography, as well as pulse pressure (PP), calculated as the difference between systolic and diastolic BP, were markers of arterial stiffness. Stiffness parameters increased significantly with the increase in ASCVD risk in the entire cohort. In 30 (14.8%) patients in the low- to moderate-risk group, the median SI was 8.07 m/s (7.10–8.73), RI 51.40% (39.40–65.60), and PP 45.50 mmHg (40.00–57.00); in 111 (54.7%) patients in the high-risk group, the median SI was 8.70 m/s (7.40–10.03), RI 57.20% (43.65–68.40), and PP 54.00 mmHg (46.00–60.75); and in 62 (30.5%) patients in the very-high-risk group, the median was SI 9.27 m/s (7.57–10.44), RI 59.00% (50.40–72.40), and PP 60.00 mmHg (51.00–67.00). In healthy participants, the SI ≤ 9.0 m/s (sensitivity of 92.31%, area under the curve [AUC] 0.686, *p* < 0.001) based on the receiver operating characteristics was the most sensitive variable for discriminating low to moderate risk, and PP > 56.0 mmHg (sensitivity of 74.36%, AUC 0.736, *p* < 0.001) was used for discriminating very high risk. In multivariate logistic regression, younger age, female sex, PP ≤ 50 mmHg, SI ≤ 9.0 m/s, and triglycerides < 150 mg/dL had the best relationship with low to moderate SCORE2 risk. In turn, older age, currently smoking, PP > 56.0 mmHg, RI > 68.6%, and diastolic BP ≥ 90 mmHg were related to very high SCORE2 risk. In conclusion, arterial stiffness is significantly related to ASCVD risk in post-COVID-19 patients and can be helpful as a single risk marker in everyday practice. Cut-off points for arterial stiffness parameters determined based on SCORE2 may help make individual decisions about implementing lifestyle changes or pharmacological treatment of ASCVD risk factors

## 1. Introduction

In high-income countries, atherosclerotic cardiovascular diseases (ASCVD) have remained the leading cause of death for many years [1]. The multimorbidity of ASCVD and other chronic illnesses, e.g., dyslipidemia, hypertension, arthritis, chronic kidney disease (CKD), and diabetes (DM), is common and increases cardiovascular (CV) risk [2]. In countries with a high risk of ASCVD, according to the World Health Organization (WHO) [3], up to one in five patients recovering from the coronavirus disease (COVID-19) had pre-existing ASCVD [4]. One in two apparently healthy post-COVID-19 individuals had a high/very high ASCVD risk due to CV-related risk factors [4]. It predisposed them to a higher risk of complications of severe acute respiratory syndrome coronavirus 2 (SARS-CoV-2) infection during the pandemic, higher mortality, and long COVID-19 syndrome [5,6,7,8,9]. It is known that post-COVID-19 patients have an increased risk of new-onset ASCVD, which persists in the post-acute phase for a year after infection, independent of major ASCVD risk factors [9,10,11,12,13]. It is a consequence of the systemic inflammatory response, chronic vascular endothelial dysfunction, damage to the endothelial barrier, increased oxidative stress, and a prothrombotic tendency [14,15,16,17]. Inflammatory cell infiltration, increased production of matrix metalloproteinases with elastin fiber degeneration, and changes in vascular smooth muscle phenotype are possible mechanisms linking inflammation to arterial stiffness [18]. One might assume that measuring arterial stiffness might be a simple way to screen the increase in ASCVD risk in the post-COVID-19 population.

Arterial stiffening is a pathological consequence of aging and the loss of vascular elasticity and is strongly associated with arteriosclerosis [19]. However, the progression of arterial stiffening is accelerated in the presence of ASCVD risk factors, such as elevated blood pressure (BP), hyperglycemia, hyperlipidemia, obesity, or systemic inflammation [18,20,21,22]. Increased arterial stiffening can be detected even before the onset of established ASCVD through the pulse wave velocity [23,24]. Furthermore, several non-invasive methods, such as photoplethysmography (PPG), offer a simple and accessible outpatient setting to measure parameters relating to the elasticity of the arterial wall with portable devices [25,26,27]. A higher stiffness index (SI) measured by PPG is associated with an increased risk of new-onset ASCVD (hazard ratio [HR] 1.27), including myocardial infarction (HR 1.38) and coronary artery disease (CAD) (HR 1.31) in 2.8 years of observation, even after adjusting to age and sex [28]. A more straightforward method of measuring arterial stiffness is pulse pressure (PP) assessment [29]. Its value as a risk predictor of overall CV disease (HR 1.57), myocardial infarction (HR 1.48), and CAD (HR 1.47) has been confirmed. PP also predicts CV mortality within 6.1 years of follow-up (HR 1.47) [28]. This ease of use and confirmation of prognostic effectiveness in a large community-based population promises the future of ASCVD risk assessment to guide preventive and diagnostic interventions.

According to the 2021 European Society of Cardiology (ESC) Guidelines on Cardiovascular Disease Prevention in Clinical Practice [30], arterial stiffness can be a valuable biomarker for risk prediction in patients close to decision-making thresholds. In contrast to risk scores based on traditional ASCVD-related risk factors, arterial stiffness can reveal the additive effect of unscaled, residual, or hidden risk factors, such as diabetes, persistent/chronic inflammation, enhanced thrombogenesis, individual genetic predisposition, or psychosocial stress [18,21,28,31,32,33,34]. However, the ESC guidelines highlight that some evidence of unreliability in publications on this topic has resulted in it not being accepted as a systematic screening method among the general population [30]. 

This makes it even more necessary to determine the feasibility of using this method as a simple, reliable, and practical screening test in various cohorts. The selection of the post-COVID-19 cohort is essential due to the widespread nature of the disease during the pandemic from 2019 to 2023. After all, every second citizen of Europe or the United States has had COVID-19 [35].

Furthermore, the ESC recommends individualizing treatment goals concerning some CV risk factors (i.e., low-density lipoprotein cholesterol [LDL-C], BP, and glycemic control) depending on the magnitude of the overall ASCVD risk, which is defined as risk classes (from low to moderate to very high). The ASCVD risk assessment, according to the ESC Systematic Coronary Risk Evaluation 2 (SCORE2) algorithm [30,36], is functional but requires a complete lipid profile to assess risk classes, precluding its ad hoc use at the first contact with healthcare services. Establishing cut-off points for arterial stiffness parameters concerning SCORE2-derived risk classes may be practical in implementing lifestyle changes or pharmacological treatment of ASCVD risk factors in apparently healthy individuals based on those parameters.

The purpose of this study was to assess the relationship of non-invasive arterial stiffness parameters with a 10-year ASCVD risk score in post-COVID-19 patients. Furthermore, this study aimed to establish cut-off points for arterial stiffness parameters to discriminate SCORE2-derived low- to moderate-risk (<5%) and very-high-risk (≥10%) participants in a healthy (in terms of ASCVD) cohort. This study assessed the usefulness of arterial stiffness parameters compared to the individual risk factors considered in CV risk estimation in daily practice, such as BP, body mass index (BMI), or lipid profile.

## 2. Materials and Methods

### 2.1. Study Group

This study was cross-sectional. It was conducted on patients in the National Health Fund (NHF) cardiac rehabilitation (CR) program up to 12 months after COVID-19 [37] hospitalized at the Cardiac Rehabilitation Department of the Ustron Health Resort, Poland. From 11 May 2021 (hospitalization of the first patient after COVID-19 at the Health Resort in Ustron) to the end of February 2022 (termination of the program by the NHF), 553 patients participated in the National Health Fund Cardiac Rehabilitation Program. Of these, 253 volunteered to participate in the preventive research project planned by the Medical University of Silesia in Katowice (Poland), while 300 did not accept the invitation. Of the 253 volunteers who consented to a medical interview, blood sampling, BP measurement, and additional examinations, including the non-invasive measurement of arterial stiffness using PPG, 231 met the inclusion criterion of age 40–69 for analysis of the relationship of non-invasive arterial stiffness parameters with ASCVD risk by ESC guidance and the SCORE2 algorithm. The cardiovascular risk of included patients at enrollment was unknown. For 28 of them, the results of arterial stiffness measurement were not reproducible, or correct laboratory analysis of the blood samples could not be performed (e.g., due to blood hemolysis). These participants were excluded from further analysis. Ultimately, 203 participants for whom complete clinical and laboratory data were available were included.

The research was approved by the Bioethics Committee of the Medical University of Silesia in Katowice (Resolution PCN/CBN/0022/KB1/68/21 on 15 June 2021 and PCN/CBN/0052/KB1/68/1/21/22 on 29 March 2022). Informed consent was obtained from all subjects involved in this study.

### 2.2. ASCVD Risk Assessment

The 2021 ESC Guidelines on Cardiovascular Disease Prevention in Clinical Practice [30] base the risk assessment on the estimation of the 10-year risk of fatal and non-fatal ASCVD events, which can be established for patients with pre-existing ASCVD/DM/CKD or apparently healthy people as low to moderate, high, and very high. 

For patients with pre-existing ASCVD/DM/CKD, the risk is estimated based on the presence of ASCVD and the severity of comorbidities. In turn, the ESC SCORE2 algorithm estimates one’s absolute 10-year risk of fatal and non-fatal ASCVD events only in apparently healthy people aged 40–69 with untreated risk factors or with risk factors that have been stable for several years. The risk is calculated in percentage based on age, sex, smoking status (current smoker vs. other), systolic BP (SBP), and non-high-density lipoprotein cholesterol (non-HDL-C) and estimated as low to moderate to very high based on cut-off points. For people 40–49, <2.5% is considered low to moderate risk, 2.5% to <7.5% is high, and ≥7.5% is a very high risk for ASCVD. For people 50–69, low to moderate risk is <5%, high risk is 5% to <10%, and very high risk is ≥10%. SCORE2 is calibrated using national ASCVD mortality rates published by the WHO [3].

Following the ESC guidelines, we categorized the study participants into three risk groups.

Group I (low- to moderate-risk group) consisted of (1) apparently healthy participants aged <50 with SCORE2 <2.5%; (2) apparently healthy participants aged 50–69 with SCORE2 <5%; and (3) participants with DM type 1 or type 2 lasting <10 years, without ASCVD/target organ damage and other CV risk factors.

Group II (high-risk group) consisted of (1) participants with DM type 2 without ASCVD/target organ damage but with other CV risk factors, i.e., obesity, hypercholesterolemia, hypertension, and CKD with an estimated glomerular filtration rate (eGFR) of 45 mL/min to <60 mL/min calculated according to the Cockcroft–Gault formula, or smoking; (2) participants with CKD with eGFR 30–44 mL/min; (3) apparently healthy participants aged <50 with SCORE2 of 2.5 to <7.5%; and (4) apparently healthy participants aged 50–69 with SCORE2 of 5 to <10%

Group III (very-high-risk group) consisted of (1) participants with pre-existing ASCVD, that is, CAD, i.e., myocardial infarction or coronary revascularization/atherosclerotic plaques in coronary artery imaging; cerebrovascular disease, i.e., a transient ischemic attack or ischemic stroke; or peripheral artery diseases with intermittent claudication or an aortic aneurysm; (2) participants with type 2 DM with established ASCVD or severe target organ damage; (3) participants with CKD with eGFR < 30 mL/min; (4) apparently healthy participants aged < 50 with SCORE2 ≥ 7.5%; and (5) apparently healthy participants aged 50–69 with SCORE2 ≥ 10%.

A participant was considered a smoker if they had actively smoked tobacco products during the year before enrollment in this study. SBP and diastolic BP (DBP) were measured using a digital electronic tensiometer (OMRON M3 Intellisense, OMRON Healthcare, Milton Keynes, United Kingdom) over the brachial artery and after a 15 min rest. Total cholesterol (TC), high-density lipoprotein cholesterol (HDL-C), LDL-C, and triglycerides (TGs) were assessed using standard enzyme methods. The concentration of non-HDL-C was calculated as the difference between TC and HDL-C.

### 2.3. Arterial Stiffness Measurement

Arterial stiffness was assessed based on pulse pressure and plethysmography. PP was defined as the mean difference (in mmHg) between SBP and DBP. PP increases with a decrease in the compliance of the large elastic arteries. The reference value for brachial PP is 50 mmHg in both men and women [38]. PP > 60 mmHg is a risk factor for CV events [39,40].

To estimate the digital volume pulse (DVP) wave by the PPG method, we used the Pulse Trace PCA2 sensor (Micro Medical Ltd., Rochester, Kent, UK). It measures the intensity of infrared waves with a length of 600–1200 nm, depending on blood perfusion [41]. The image of the DVP curve reflects the shape of the pulse wave and consists of two components: the early systolic component (“a” wave) and the diastolic component (“b” wave). The “a” wave results from the direct propagation of the pulse wave from the aorta to the finger, while the “b” wave is a product of overlapping the return flow of the wave reflected from the resistance arterioles and transmitted again toward the lower body [25,42]. The measurements were carried out using a sensor on the supine subjects’ index fingers. The mean value from five measurements was used for statistical analysis.

The parameters used to assess the elasticity of arterial walls were as follows [43]:

- Peak-to-peak time (PPT, m/s) indicates the time elapsed from the systolic to the diastolic peak of the DVP wave;
- The stiffness index (SI, m/s) reflects the time between the peak systolic and diastolic wave and is indexed to the subject’s height (calculated as the height divided by the PPT time);
- The reflection index (RI, %) assesses what percentage of the systolic wave amplitude is the diastolic wave amplitude (calculated as the “b” wave amplitude divided by the “a” wave amplitude and multiplied by 100%).

The PPT and the SI correlate with pulse wave propagation velocity and reflect stiffness within large arteries. The speed of the pulse wave increases proportionately to the increase in vascular stiffness (the shorter the PPT and the higher the SI value, the greater the arterial stiffness). The RI assesses the tone of small- and medium-sized arteries. In stiff vessels, the reflection point of the pulse wave occurs closer to the heart. It creates a strong reflection wave that adds to the ejection wave and leads to an unfavorable increase in systolic pressure in the aorta and left ventricular overload (the higher the RI value, the greater the arterial stiffness) [25,42]. The SI values of 8.8 ± 2.8 m/s assessed using Pulse Trace PCA2 have been described as average values for a large group of healthy middle-aged participants [44]. For the cohort of younger healthy volunteers (age approximately 35 ± 12 years) tested using the same method, the SI was 8.11 ± 2.21 m/s for women and 8.26 ± 2.13 m/s for men, while the RI was 74 ± 11% and 73 ± 14%, respectively [42].

### 2.4. Statistical Analysis

The results were analyzed using MedCalc Version 22.021 (MedCalc Software Ltd., Ostend, Belgium). The Kolmogorov–Smirnov test assessed the normality of the distribution. The quantitative parameters were presented as the median and lower and upper quartile. The nonparametric Kruskal–Wallis test with the Conover post hoc test and the Jonckheere–Terpstra test for trends were used as the methods of choice to compare values of arterial stiffness and other CV risk factors by ASCVD risk classes and demonstrate how those values changed with increasing risk according to the SCORE2 classification. Qualitative data were expressed as numbers and percentages (%) with differences in the frequency of arterial stiffness risk factors by ASCVD risk classes calculated using the chi-squared test with the Cochran–Armitage test for trends. The relationship between arterial stiffness parameters and the 10-year risk of fatal and non-fatal ASCVD events according to SCORE2 was evaluated by linear regression. A receiver operating characteristics (ROC) curve was applied to establish the optimal cut-off points to discriminate SCORE2-derived low to moderate (<5%) and very high (≥10%) ASCVD risk participants in apparently healthy individuals. The usefulness of these cut-off points was compared in the univariable and multivariable logistic regression with the single parameters and cut-off points taken into account in risk estimation in everyday practice. A two-sided *p*-value less than 0.05 was considered statistically significant.

During this study, a power analysis was performed based on differences in selected continuous variables (e.g., PP and SI level) between the three analyzed groups using a one-way analysis of variance with a significance level of 0.05. For these continuous parameters, over 90% of the power was reported.

## 3. Results

### 3.1. General Characteristics of the Study Group

The study group comprised 203 post-COVID-19 participants aged 60.0 (55.0–63.0) and 115 (56.7%) women.

Based on medical records, we determined the severity of the acute phase of COVID-19 in 198 out of 203 patients, following the guidelines of the Polish Society of Epidemiologists and Infectiologists [45]. COVID-19 severity was mild in 101 patients (51.0%). These patients were asymptomatic or mildly symptomatic, with an arterial oxygen saturation (SpO2) of ≥94% in room air, without needing oxygen therapy or hospitalization due to infection. In 55 patients (27.8%), COVID-19 severity was moderate. These participants were fully symptomatic, with an SpO2 of 90–94% in room air; nine of them (16.4%) required home oxygen therapy, and thirty-eight (69.1%) were hospitalized, predominately due to concomitant pneumonia (52 participants, 94.5%). Severe COVID-19 was diagnosed in twenty-eight patients (14.1%) with respiratory failure/desaturation of <90% in room air or the involvement of ≥50% of the lung on computed tomography (four participants, 14.3%) or with pulmonary embolism (four participants, 14.3%). All patients in this subgroup required hospitalization. Fourteen (50.0%) participants in the severe COVID-19 group had high-flow nasal oxygen therapy or non-invasive ventilation. In the group of fourteen patients (7.1%), COVID-19 was stratified as critical due to septic shock (five participants, 37.5%) or due to the need for intensive care unit treatment caused by multi-organ failure (fourteen participants, 100.0%) or severe respiratory failure/acute respiratory distress syndrome (ten participants, 71.4%) with invasive ventilation. 

The current smokers group consisted of 21 patients (10.3%). The average duration of smoking in this subgroup was as long as 25.62 ± 11.56 years. The former smokers comprised 70 patients (34.5%). The average duration of smoking in this subgroup was 21.66 ± 11.28 years, but the time since quitting was, on average, 17.01 ± 10.04 years. The never-smoker group consisted of 112 patients (55.2%). At the time of enrollment, only one patient smoked e-cigarettes, but he had smoked traditional tobacco products before and was counted with other smokers.

Table 1 and Table 2 present the general characteristics, arterial stiffness, and lipid profile of the entire post-COVID-19 cohort and subgroups based on ASCVD risk. We classified 30 (14.8%) patients in the low- to moderate-risk group, 111 (54.7%) in the high-risk group, and 62 (30.5%) in the very high ASCVD risk group. 

### 3.2. Relationship of the Arterial Stiffness Parameters with ASCVD Risk in the Entire Cohort

The prevalence of comorbidities, as well as the participant’s age, BMI, SBP, and non-HDC-C level, increased in parallel with increases in estimated ASCVD risk. The proportion of men and smokers in the groups was also higher in the higher-risk groups. Among the parameters assessing arterial stiffness, an increase in the SI, the RI, and PP was found to be proportional to the ASCVD risk. In the entire group, elevated PP > 60 mmHg had sixty-one participants (30.0%), including twenty-seven (44.3%) from the very-high-risk group and twenty-eight (45.9%) from the high-risk group, but only six (9.8%) from the low- to moderate-risk group, *p* = 0.018. Despite the bias caused by the routine use of hypertensives in patients with high/very high risk, the values of arterial stiffness parameters increased in proportion to the risk of ASCVD.

### 3.3. Relationship of the Arterial Stiffness Parameters with the SCORE2 Algorithm in the Healthy Group

For 141 apparently healthy (in terms of ASCVD) participants, we determined the individual 10-year fatal and non-fatal ASCVD-related events risk in percentages based on the SCORE2 algorithm. For participants aged 40–49, the median risk was 3.0% (2.0–4.0): 2.0% (1.0–2.0) in the low- to moderate-risk group and 4.0% (3.0–5.3) in the high-risk group. None of the subjects aged 40–49 had a very high ASCVD risk. In the group of participants aged 50–69, the median risk was 8.0% (5.0–10.0), including 4.0% (3.0–4.0) in the low- to moderate-risk group, 7.0% (6.0–8.0) in the high-risk group, and 11.0% (10.0–14.0) in the very-high-risk group. The SI, the RI, and PP values increased linearly as the SCORE2 risk increased; *p* < 0.001 for all parameters. The median SCORE2 risk in the subgroup with PP > 60 mmHg was 9.0% (5.25–11.75) vs. 7.0% (4.0–9.0) among the others; *p* = 0.002.

### 3.4. Usefulness of Arterial Stiffness Cut-Off Points Compared to the Individual Classic Risk Factors in the Healthy Group

An ROC curve was applied to establish the optimal cut-off points to determine participants’ ASCVD risk as <5% (for statistical purposes, this was assumed as low to moderate for the entire cohort, regardless of participants’ age) and ≥10% (assumed as very high) in the apparently healthy group. The ROC analysis showed that the SI ≤ 9.0 m/s (sensitivity of 92.31%, AUC 0.686, *p* < 0.001) was the most sensitive variable for discriminating participants with a low- to moderate risk of ASCVD. This parameter was also suitable for discriminating patients with a very high risk, with a sensitivity of 64.10% for the SI > 9.0 m/s (AUC 0.687, *p* < 0.001). However, PP > 56.0 mmHg better discriminated the very-high-risk group (sensitivity of 74.36%, AUC 0.736, *p* < 0.001) than the SI, followed by the low- to moderate-risk group (sensitivity of 61.54%, AUC 0.649, *p* = 0.005). The sensitivity of the RI was less suitable for the ASCVD risk assessment (for a low- to moderate-risk estimation sensitivity of 58.97%, AUC 0.622, *p* = 0.022; for a very-high-risk estimation sensitivity of 43.59%, AUC 0.625, *p* = 0.020).

The usefulness of these cut-off points was compared in the univariable and multivariable logistic regression with the single parameters and cut-off points taken into account in risk estimation in everyday practice in the healthy population (i.e., BMI < 25.0 kg/m^2^ vs. ≥ 30.0 kg/m^2^, BP < 120/80 mmHg vs. ≥ 140/90 mmHg, LDL-C 115.0 mg/dL, non-HDL-C 145 mg/dL, TG 150 mg/dL, and TC 190 mg/dL), as shown in Table 3 and Table 4.

In multivariate analysis, younger age, female sex, PP ≤ 50 mmHg, SI ≤ 9.0 m/s, and TG < 150 mg/dL had the best relationship with low to moderate SCORE2 risk. In turn, older age, currently smoking, PP > 56.0 mmHg, RI > 68.6%, and DBP ≥ 90 mmHg were related to very high SCORE2 risk. Cut-off points for BMI, SBP, LDL-C, non-HDL-C, TGs, and TC were not found to be independently associated with SCORE2 cut-off points.

## 4. Discussion

This study evaluated the relationship of non-invasive arterial stiffness parameters with an individual 10-year risk of fatal and non-fatal ASCVD events in the post-COVID-19 cohort. In the entire cohort, we found that the change in the SI and PP (reflecting stiffness within large arteries) was proportional to ASCVD risk. Moreover, we confirmed the linear relationship between the SI and PP and ASCVD risk expressed in percentages based on the new SCORE2 algorithm in a group of healthy (in terms of ASCVD) participants. In ROC analysis, the SI with a cut-off value of ≤9.0 m/s had very sensitive variables to discriminate low- to moderate-risk (<5%) participants. It was also suitable for discriminating very high risk (≥10%) with a cut-off point of >9.0 m/s. However, PP > 56.0 mmHg better discriminated the very-high-risk group than the SI. Concerning the RI (reflecting the tone of small- and medium-sized arteries), the parameter also increased proportionally to ASCVD risk in the entire cohort, and a linear relationship was confirmed between this parameter and the risk expressed in percentages in healthy participants. However, the sensitivity of the RI was less suitable for risk assessment. In multivariate logistic regression, younger age, female sex, PP ≤ 50 mmHg, SI ≤ 9.0 m/s, and TG < 150 mg/dL had the best relationship with low to moderate SCORE2 risk. In turn, older age, currently smoking, PP > 56.0 mmHg, RI > 68.6%, and DBP ≥ 90 mmHg were related to very high SCORE2 risk. Stiffness parameters (i.e., the SI and PP) better discriminate the low to moderate ASCVD risk group than optimal SBP, correct BMI, and normal lipid parameters (except for TG concentration), which are considered in risk estimation in everyday practice. Similarly, the RI and PP are more valuable than the male sex, SBP ≥ 140 mmHg, BMI ≥ 30.0 kg/m^2^, TC ≥ 190 mg/dL, and LDL ≥ 115 mg/dL in discriminating the very high risk ASCVD group in healthy participants. Thus, SCORE2-derived cut-off points for arterial stiffness parameters may be practical in setting goals for risk factor management as a reasonable alternative to the SCORE2 algorithm at the first contact with healthcare services.

Among Polish post-COVID-19 patients, 13.5% have a history of ASCVD (the equivalent of very high ASCVD risk), half have elevated BP or non-HDL-C values, 27% have DM, and approximately 10% smoke cigarettes. Even among apparently healthy convalescents, the percentage of those with high/very high ASCVD risk is significant [4]. The high burden of cardiovascular diseases translates into the high mortality rate reported by the WHO in our country [3]. Even in the Netherlands, a country with low ASCVD risk, according to the WHO [3], no less than 5.6% of the post-COVID-19 cohort still have a history of ASCVD. However, the prevalence of ASVCD risk factors is lower and estimated at 20% for hypertension, 15% for dyslipidemia, and 10% for diabetes (but 20% for currently smoking). Due to adverse lifestyle alterations during a pandemic, a disturbing increase in the prevalence of acquired ASCVD-related risk factors is observed in the general population, even adolescents [46,47]. It has prompted the search for methods assessed in this population to facilitate individualizing preventive and therapeutic measures.

To date, no studies have assessed the relationship of the PPG measurements in terms of SCORE2 or other CV risk scales in a cohort of post-COVID-19 convalescents. However, other researchers have verified the predictive value of PPG against CV risk scales in different patient groups.

Gunarathne et al. [26] investigated the predictive value of arterial stiffness parameters measured by PPG using the PCA 2 Micro Medical device based on ESC HeartScore [48]. The study was conducted in a pre-COVID-19 cohort of participants with ASCVD risk factors and a healthy subgroup. ESC HeartScore estimates a 10-year risk only for a fatal CV event, taking into account age, sex, SBP, smoking, and TC (but not a non-HDL-C). In this study, as in ours, the SI increased from low- to moderate-risk to very-high-risk participants and was strongly associated with ASCVD risk factors, such as hypertension, DM, hypercholesterolemia, and cigarette smoking, but not BMI. Moreover, as in our healthy subgroup, the SI was proportional to ASCVD risk scores on a linear regression analysis. The authors noted that the SI was more discriminative between low to medium and high-risk categories when compared to TC, plasma glucose, SBP, and waist to hip ratio. 

Tąpolska et al. [49] assessed the correlation between the SI and the RI concerning an individual 10-year CV disease risk calculated using the Heart Risk Calculator (RISK) algorithm by the American Heart Association [50]. Measurements were performed using the Pulse Trace PCA 2 apparatus in a pre-COVID-19 cohort aged 59.02 ± 9.24 without pre-existing ASCVD. Unlike ESC SCORE2, AHA RISK considers TC and HDL-C (instead of non-HDL-C) and the presence of diabetes. The authors found the most robust relationship between the RISK score and the SI for women, participants aged 40–54 with normal BMI, and between the RISK score and the RI for participants aged 40–54 with BMI > 30 kg/m^2^. As in our study, the SI was rated as more valuable than the RI in predicting the individual risk of future CV events. 

Said et al. [28] examined arterial stiffness using PulseTrace PCA2 and calculated PP in a large cohort of 169,613 UK Biobank participants (mean age 56.8 years). The prevalence and incidence of CV risk factors and events were assessed using patient-reported data. The value of arterial stiffness parameters correlated well with the risk of new-onset ASCVD and all-cause and CV mortality. What was particularly important was that the study confirmed that the SI measurement improved the risk prediction model by 2.3% within 5.9 years when added to the Framingham Risk Score [51]. PP rating improves it even further by 5.4%. The authors concluded that a risk scale based solely on classic risk factors may underestimate the risk of ASCVD in the presence of additional burdens. Assessment of arterial stiffness does not have this disadvantage and may improve risk prediction. As in our study, PP discriminated against the high-/very-high-risk group against the participants with low/moderate risk better than the SI.

An example of assessing the correlation of arterial stiffness with the CV risk score in chronic inflammatory diseases is the study by Triantafyllias et al. [52]. The authors analyzed aortic stiffness measured by carotid-femoral pulse wave velocity (cfPWV) compared with ESC HeartScore [48] in a cohort with psoriatic arthritis. Inflammatory rheumatic diseases are known to be associated with increased CV risk and mortality [21]. The authors confirmed elevated aortic stiffness and end-organ disease in 16.0% of participants. The cfPWV was independently associated with disease duration and traditional ASCVD-related risk factors such as age, high BP, and CKD. In the same cohort, SCORE revealed an elevated CV risk in only 8.7% of patients, thus underestimating the risk increase by chronic inflammation. Our cohort had no significant discrepancy between the risk assessed based on arterial stiffness compared to estimation based on the occurrence of risk factors or the SCORE2 algorithm. The median SCORE2 risk in the subgroup with PP elevated above 60 mmHg (indicating an increased ASCVD risk) was 9.0% (close to 10%, indicating very high risk in the SCORE2) compared to 7.0% among the others. In the entire cohort, elevated PP was observed in 44.3% with very high ASCVD risk but only in 9.8% of participants with low to moderate risk. However, we did not determine the percentage of participants with increased SI and RI values because no standard references/cut-off points to these parameters for PPG have been adopted. It can be assumed that the inflammatory component in post-COVID-19 convalescents is much less severe than in primary inflammatory diseases or lasts too short to accelerate arteriosclerosis.

It is worth emphasizing that evaluating arterial stiffness and other ASCVD risk factors together may provide even better predictive values than evaluating individual risk factors. Wu et al. [33] assessed arterial stiffness parameters (using brachial ankle pulse wave velocity), glycemic control, and BP values as risk factors for macrovascular complications in type 2 DM. Patients with both severe arterial stiffness and poor glycemic control (HR 2.73) or hypertension (HR 2.69) had a higher risk of macrovascular complications than those with a single risk factor. Niiranen et al. [32] assessed cfPWV and classical CV risk factors in 2127 community-dwelling participants of the Framingham Offspring Cohort. High cfPWV was associated with a trend toward increasing CV risk in non-hypertensives and hypertensives in a median 12.6-year follow-up. CV risk was higher in normotensives with high cfPWV (HR 1.29) than in normotensives with low cfPWV and in hypertensives with high cfPWV (HR 2.25) than in hypertensives with low cfPWV (HR 1.54). The authors note that arterial stiffness may explain a residual CV risk associated with well-controlled hypertension. Due to the lack of follow-ups, we could not make such observations in our group. However, the PPG-derived SI and the calculated PP were more valuable than BP cut-off points in predicting very high and low to moderate ASCVD risk, according to SCORE2.

### Study Limitations

Due to the end of the post-COVID-19 rehabilitation program that the NHF implemented in Poland, we could not collect a more extensive study group. Furthermore, post-COVID-19 patients participating in the NHF program came from different, often remote, parts of Poland, which made follow-up challenging. We based the assessment of the frequency of comorbidities on medical interviews and available documentation, which may not always adequately reflect the actual rates of diseases. In particular, the percentage of participants with peripheral arterial disease (often without overt symptoms) is meager, although up to 20% of people with this disease are expected to be in the age group between 55 and 75 years [53]. Moreover, the percentage of patients with CAD is relatively small. It cannot be ruled out that a particular group of patients with exacerbation of CAD in the course of COVID-19, such as myocardial infarction or acute coronary syndrome/coronary revascularization, could have been referred for CR under the Polish Managed Care after the Acute Myocardial Infarction program run in parallel by the NHF. The assessed method for estimating arterial stiffness was the PPG. We did not verify the measurement results against cfPWV (the gold standard for the non-invasive assessment of arterial stiffness) or central PP measured at the carotid artery [24]. We did not determine the percentage of participants with normal/increased SI and RI values because no references to PPG parameters had been adopted. Because we assessed the risk of atherosclerotic complications, we focused on parameters of large artery stiffness and did not analyze the changes in the RI under the influence of beta 2-adrenergic vasodilators for determining endothelium function [54]. Since 174 (85.7%) of the respondents were ≥50 years old, we assumed a low to moderate ASCVD risk of <5% and a very high risk of ≥10% for all participants and used these values in the ROC analysis to establish the optimal cut-off points for ASCVD risk factors.

## 5. Conclusions

Arterial stiffness is significantly related to the ASCVD risk in post-COVID-19 patients and can be helpful as a single risk marker in everyday practice. The SI, the RI, and PP can better discriminate between low to moderate and very high ASCVD risk groups than individual classic risk factors such as SBP, lipid profile, and BMI. Thus, SCORE2-derived cut-off points for arterial stiffness parameters may be practical for making individual decisions about implementing lifestyle changes or pharmacological treatment for ASCVD risk factors.

## Figures and Tables

**Table 1:** General characteristics of the entire post-COVID-19 cohort and subgroups based on atherosclerotic cardiovascular disease-related events risk.

Parameters		All Participants 203 (100.0%)	ASCVD-Related Events Risk Groups			*p*-Value for Trend
			Low/Moderate (1) 30 (14.8%)	High (2) 111 (54.7%)	Very High (3) 62 (30.5%)	
Age (years), M (lower–upper quartile)		60.0 (55.0–63.0)	51.0 (47.0–58.0)	59.0 (54.0–62.0)	62.0 (60.0–65.0)	<0.001 (1) ≠ (2) (3) (2) ≠ (3)
Sex, *n* (%)	men	88 (43.3%)	4 (13.3)	50 (45.0)	34 (54.8)	<0.001 (1) ≠ (2) (1) ≠ (3)
	women	115 (56.7%)	26 (86.7)	61 (55.0)	28 (45.2)	
BMI (kg/m^2^), M (lower–upper quartile)		29.00 (26.05–32.31)	26.10 (24.22–30.06)	29.40 (26.44–31.86)	29.84 (26.22–33.57)	0.019 (1) ≠ (2) (3)
Obesity, *n* (%)		89 (43.8)	8 (26.7)	51 (45.9)	30 (48.4)	0.085
Pre-existing ASCVD, *n* (%)		14 (6.9)	0.0 (0.0)	0.0 (0.0)	14 (22.6)	<0.001 (1) (2) ≠ (3)
Heart failure, *n* (%)		13 (6.4%)	2 (6.7)	6 (5.4)	5 (8.1)	0.677
Chronic pulmonary disease, *n* (%)		22 (10.8)	8 (26.7)	8 (7.2)	6 (9.7)	0.086
Atrial fibrillation, *n* (%)		6 (3.0)	1 (3.3)	3 (2.7)	2 (3.2)	0.973
Hypertension, *n* (%)		122 (60.1)	8 (26.7)	70 (63.1)	44 (71.0)	0.002 (1) ≠ (2) (1) ≠ (3)
Diabetes, *n* (%)		44 (21.7)	0 (0.0)	37 (33.3)	7 (11.3)	0.987 (1) ≠ (2) (2) ≠ (3)
Chronic kidney disease *, *n* (%)		9 (4.4)	1 (3.3)	4 (3.6)	4 (6.5)	0.375
Dyslipidemia ^#^, *n* (%)		106 (52.2)	9 (30.0)	61 (55.0)	36 (58.1)	0.027 (1) ≠ (2) (1) ≠ (3)
Current smokers, *n* (%)		21 (10.3)	0 (0.0)	10 (9.0)	11 (17.7)	0.008 (1) ≠ (3)
Beta blockers		93 (45.8)	12 (40.0)	48 (43.2)	33 (53.2)	0.143
ACE-I/ARB		72 (35.5)	3 (10.0)	38 (34.2)	31 (50.0)	<0.001 (1) ≠ (2) (3) (2) ≠ (3)
MRA		9 (4.4)	0 (0.0)	2 (1.8)	7 (11.3)	0.004 (1) ≠ (3) (2) ≠ (3)
Loop diuretics		10 (4.9)	1 (3.3)	6 (5.4)	3 (4.8)	0.818
Acetylsalicylic acid		38 (18.7)	3 (10.0)	12 (10.8)	23 (37.1)	<0.001 (1) ≠ (3) (2) ≠ (3)

* With creatinine clearance < 60 mL/min; ^#^ with total cholesterol ≥ 190 mg/dl, low-density lipoprotein cholesterol ≥ 115 mg/dl or taking lipid-lowering medications; ≠ : difference (*p* < 0.05) between groups in the Conover post hoc test; ACE-I: angiotensin-converting enzyme inhibitors; ARB: angiotensin receptor blocker; ASCVD: atherosclerotic cardiovascular disease (coronary artery disease, cerebrovascular disease, or peripheral artery disease); BMI: body mass index; chronic pulmonary disease: asthma, emphysema, chronic obstructive pulmonary disease; M: median; MRA: mineralocorticoid-receptor antagonists; *n*: number.

**Table 2:** The arterial stiffness parameters and lipid profile of the entire post-COVID-19 cohort and subgroups based on atherosclerotic cardiovascular disease-related events risk.

Parameters	All Participants 203 (100.0%)	ASCVD-Related Events Risk Groups			
		low/Moderate (1) 30 (14.8%)	High (2) 111 (54.7%)	Very High (3) 62 (30.5%)	*p*-Value for Trend
SI (m/s), M (lower–upper quartile)	8.62 (7.40–9.96)	8.07 (7.10–8.73)	8.70 (7.40–10.03)	9.27 (7.57–10.44)	0.003 (1) ≠ (2) (1) ≠ (3)
RI (%), M (lower–upper quartile)	57.80 (44.53–68.60)	51.40 (39.40–65.60)	57.20 (43.65–68.40)	59.00 (50.40–72.40)	0.026
SBP (mmHg), M (lower–upper quartile)	131.00 (119.00–144.25)	122.00 (112.00–133.00)	132.00 (116.25–144.75)	133.50 (125.00–146.50)	0.005 (1) ≠ (2) (1) ≠ (3)
DBP (mmHg), M (lower–upper quartile)	80.00 (75.00–85.00)	77.00 (70.00–82.00)	80.00 (75.00–87.00)	80.00 (76.00–85.00)	0.079
PP (mmHg), M (lower–upper quartile)	55.00 (46.00–65.00)	45.50 (40.00–57.00)	54.00 (46.00–60.75)	60.00 (51.00–67.00)	<0.001 (1) ≠ (2) (3) (2) ≠ (3)
Creatinine (mg/dL), M (lower–upper quartile)	0.93 (0.85–1.03)	0.85 (0.83–0.97)	0.95 (0.86–1.07)	0.95 (0.85–1.02)	0.098 (1) ≠ (2) (1) ≠ (3)
Fasting glucose (mg/dL), M (lower–upper quartile)	87.15 (80.40–97.10)	80.75 (75.80–84.90)	89.20 (81.60–99.88)	88.70 (80.60–97.18)	0.018 (1) ≠ (2) (1) ≠ (3)
CRP (mg/dL), M (lower–upper quartile)	2.30 (1.30–4.73)	2.10 (1.10–4.40)	2.30 (1.33–4.68)	2.35 (1.35–4.85)	0.429
TC (mg/dL), M (lower–upper quartile)	228.70 (192.50–277.58)	225.15 (192.80–275.90)	223.50 (192.20–272.63)	241.15 (192.55–284.75)	0.419
HDL-C (mg/dL), M (lower–upper quartile)	63.80 (49.08–82.48)	73.75 (58.40–91.30)	61.10 (46.48–77.55)	62.40 (45.25–89.20)	0.131 (1) ≠ (2) (1) ≠ (3)
LDL-C (mg/dL), M (lower–upper quartile)	142.95 (118.60–171.10)	146.95 (116.00–171.10)	139.00 (118.83– 169.70)	154.30 (119.40–177.30)	0.429
TG (mg/dL), M (lower–upper quartile)	169.80 (126.00–229.93)	126.75 (102.00–154.80)	182.40 (135.08–246.45)	170.40 (136.450–225.30)	0.012 (1) ≠ (2) (1) ≠ (3)
Non-HDL-C (mg/dL), M (lower–upper quartile)	165.90 (130.78–201.40)	143.65 (124.70–184.10)	168.10 (132.95–196.50)	174.90 (133.65–213.60)	0.031

≠: Difference (*p* < 0.05) between groups in the Conover post hoc test; CRP: C-reactive protein; DBP: diastolic blood pressure; HDL-C: high-density lipoprotein cholesterol; LDL-C: low-density lipoprotein cholesterol; M: median; *n*: number; non-HDL-C: non-high-density lipoprotein cholesterol; PP: pulse pressure; RI: reflection index; SBP: systolic blood pressure; SI: stiffness index; TC: total cholesterol; TGs: triglycerides. The conversion factor to SI units for glucose is [in mg/dL] × 0.0551. The conversion factor to SI units for serum creatinine concentration is [in mg/dL] × 88.42. mg/dL. The conversion factor to SI units for CRP is [in mg/dL] × 95.2. The conversion factors to SI units for TC, HDL-C, and LDL-c are [in mg/dL] × 0.02586. The conversion factor to SI units for triglycerides is [in mg/dL] × 0.01129.

**Table 3:** Relationship of arterial stiffness and other cardiovascular risk parameters with low to moderate atherosclerotic cardiovascular disease-related event risk according to the Systematic Coronary Risk Evaluation 2 algorithm in the apparently healthy cohort.

Parameters with the Optimal Criterion Value	Relationship with Low/Moderate ASCVD-Related Event Risk According to the SCORE2 Algorithm in the Apparently Healthy Cohort					
	Full Model			Stepwise Regression *p* < 0.001; Nagelkerke R^2^: 0.684		
	Coefficient	OR (95% CI)	*p*-Value	Coefficient	Standard Error	*p*-Value
Age (years)	0.247	1.28 (1.179–1.391)	<0.001	0.325	0.066	<0.001
Sex (woman)	−0.788	0.45 (0.201–1.031)	0.059	−1.773	0.785	0.024
Smoking status (never smoker)	−0.928	0.40 (0.170–0.918)	0.031	not significant in the model		
PP ≤ 50 (mmHg)	−1.393	0.25 (0.114–0.539)	<0.001	−1.862	0.636	0.003
SI ≤ 9.0 (m/s)	−2.169	0.11 (0.038–0.345)	<0.001	−1.633	0.811	0.044
RI ≤ 54 (%)	−0.969	0.38 (0.178–0.808)	0.012	not significant in the model		
SBP < 120 (mmHg)	−0.439	0.64 (0.297–1.400)	0.267	not included in the model		
DBP < 80 (mmHg)	−0.649	0.52 (0.246–1.112)	0.092	not significant in the model		
BMI < 25.0 (kg/m^2^)	−0.346	0.71 (0.297–1.687)	0.435	not included in the model		
TC < 190 (mg/dL)	−0.304	0.74 (0.287–1.896)	0.528	not included in the model		
LDL-C < 115 (mg/dL)	−0.461	0.63 (0.241–1.649)	0.347	not included in the model		
TG < 150 (mg/dL)	−0.921	0.40 (0.187–0.848)	0.017	−1.653	0.666	0.013
Non-HDL-C < 145 (mg/dL)	−1.260	0.28 (0.130–0.620)	0.002	not significant in the model		

BMI: body mass index; non-HDL-C: non-high-density lipoprotein cholesterol; LDL-C: low-density lipoprotein cholesterol; PP: pulse pressure; RI: reflection index; SBP: systolic blood pressure; SI: stiffness index; TC: total cholesterol; TGs: triglycerides.

**Table 4:** Relationship of arterial stiffness and other cardiovascular risk parameters with very high atherosclerotic cardiovascular disease-related event risk according to the Systematic Coronary Risk Evaluation 2 algorithm in the apparently healthy cohort.

Parameters with the Optimal Criterion Value	Relationship with Very High ASCVD-Related Event Risk According to the SCORE2 Algorithm in the Apparently Healthy Cohort					
	Full Model			Stepwise Regression *p* < 0.001; Nagelkerke R^2^: 0.7235		
	Coefficient	OR (95% CI)	*p*-Value	Coefficient	Standard Error	*p*-Value
Age (years)	0.304	1.36 (1.198–1.532)	<0.001	0.487	0.106	<0.001
Sex (man)	0.598	1.82 (0.860–3.847)	0.118	not significant in the model		
Smoking status (current smoker)	1.088	2.97 (0.967–9.114)	0.057	2.987	1.095	0.006
PP >56.0 (mmHg)	1.714	5.55 (2.428–12.692)	<0.001	3.090	0.752	<0.001
SI > 9.0 (m/s)	1.455	4.29 (1.963–9.356)	<0.001	not significant in the model		
RI > 68.6 (%)	1.033	2.81 (1.276–6.188)	0.010	1.714	0.735	0.020
SBP ≥ 140 (mmHg)	0.972	2.64 (1.223–5.710)	0.013	not significant in the model		
DBP ≥ 90 (mmHg)	0.943	2.57 (0.968–6.807)	0.058	2.668	0.930	0.004
BMI ≥ 30.0 (kg/m^2^)	0.306	1.36 (0.641–2.876)	0.425	not included in the model		
TC ≥ 190 (mg/dL)	0.467	1.60 (0.551–4.620)	0.390	not included in the model		
LDL-C ≥ 115 (mg/dL)	0.331	1.39 (0.476–4.077)	0.546	not included in the model		
TG ≥ 150 (mg/dL)	0.734	2.08 (0.935–4.640)	0.073	not significant in the model		
Non-HDL-C ≥ 145 (mg/dL)	1.173	3.23 (1.237–8.436)	0.017	not significant in the model		

BMI: body mass index; non-HDL-C: non-high-density lipoprotein cholesterol; LDL-C: low-density lipoprotein cholesterol; PP: pulse pressure; RI: reflection index; SBP: systolic blood pressure; SI: stiffness index; TC: total cholesterol; TGs: triglycerides.

## Data Availability

The raw data supporting the study’s conclusions will be openly available in the Polish Platform of Medical Research under the CC BY license. https://ppm.sum.edu.pl/info/researchdata/SUM26acfedb02e44df4aaa2b1d4a52c532a/; URN urn:umed-kat-prod:SUM26acfedb02e44df4aaa2b1d4a52c532a.
